# The correlation between mobile phone addiction and coping style among Chinese adolescents: a meta-analysis

**DOI:** 10.1186/s13034-021-00413-2

**Published:** 2021-10-15

**Authors:** Guang-Li Lu, Yue-Ming Ding, Yi-Ming Zhang, Hai-Tao Huang, Yi-Pei Liang, Chao-Ran Chen

**Affiliations:** 1 Institute of Business Administration, School of Business, Henan University, Kaifeng, Henan China; 2 Institute of Nursing and Health, School of Nursing and Health, Henan University, Jinming Avenue, Kaifeng, 475004 Henan China

**Keywords:** Mobile phone addiction, Coping style, Adolescent, Meta-analysis

## Abstract

**Background:**

Mobile phone addiction has become a social problem that affects the healthy growth of adolescents, and it may be correlated with coping style. The aim of this study was to investigate the relationship between mobile phone addiction and coping style and the influencing factors for adolescents.

**Methods:**

A meta-analysis was conducted by searching China National Knowledge Infrastructure (CNKI), WANFANG DATA and Chongqing VIP Information Co., Ltd. (VIP), PubMed, Web of Science, Embase, and PsycINFO. Stata 16.0 was used to analyse the overall effect and test the moderating effect.

**Results:**

Thirty-three studies were included, involving a total of 20,349 subjects. There was no significant correlation between adolescents’ mobile phone addiction and positive coping style (*r* =  − 0.02, 95% CI =  − 0.06 to 0.02, *P* > 0.05), but there was a moderate positive correlation between adolescents’ mobile phone addiction and negative coping style (*r* = 0.31, 95% CI = 0.26 to 0.36, *P* < 0.001). The moderating effect analysis showed that the effect of dissertations on mobile phone addiction and positive coping style among adolescents was significantly larger than that of journal articles. The Smartphone Addiction Scale for College Students (SAS-C) showed the largest effect on mobile phone addiction and positive coping style among adolescents. The time of publication significantly positively moderated the relationship between mobile phone addiction and negative coping style among adolescents. The Simplified Coping Style Questionnaire (SCSQ) showed the largest effect on adolescents’ mobile phone addiction and negative coping style. However, the correlation between adolescents’ mobile phone addiction and coping style was not affected by age or gender.

**Conclusions:**

There was a close relationship between mobile phone addiction and coping style among adolescents. In the future, longitudinal research should be carried out to better investigate the dynamic changes in the relationship between mobile phone addiction and coping style.

**Supplementary Information:**

The online version contains supplementary material available at 10.1186/s13034-021-00413-2.

## Background

In recent years, with the rapid development of information technology, mobile phones have been integrated as an indispensable part of people’s daily lives, with extremely rich functions. Their utilization rate is also increasing. According to the 47th China statistical report on Internet development, by the end of December 2020, China had 986 million mobile phone users, accounting for 99.7% of the total number of Internet users in China [1]. Although mobile phones make daily life more convenient, frequent use can also lead to mobile phone addiction [2, 3]. Adolescents are in a critical period of behaviour change [4], and their low level of self-control and strong curiosity about new things make it easy for mobile phone use to lead to problems [5, 6]. At present, mobile phone addiction has become a social problem that affects the healthy growth of adolescents. Relevant studies have shown that the prevalence of mobile phone addiction among Chinese adolescents exceeds 25% [7], which seriously endangers their physical health [8, 9], mental health [10, 11], academic performance [9, 12, 13] and social relations [14]. Therefore, to reduce the level of adolescents’ mobile phone addiction, researchers have actively explored the causes and mechanisms of this addiction, and found that coping style constitutes an important external factor affecting this type of addiction in adolescents [15–17].

However, many researchers have drawn inconsistent conclusions about the relationship between coping style and adolescents’ mobile phone addiction. Most studies have shown that there is a significant positive correlation between negative coping style and mobile phone addiction among adolescents. For example, He et al. [17] found that adolescents with high levels of loneliness were more inclined to adopt negative coping style, which leads to more serious problems with mobile phone addiction. However, other researchers found a significant negative correlation between negative coping style and mobile phone addiction among adolescents. For example, Quan et al. [18] found that adolescents who adopt negative coping style usually adjust their emotions by staying alone rather than using mobile phones, thus having a low level of mobile phone addiction. Similarly, there are several views on the relationship between positive coping style and mobile phone addiction among adolescents. Some studies found a significant negative correlation between them. For example, Chen et al. [19] found that positive coping styles such as problem solving and help seeking can inhibit mobile phone addiction in adolescents. Some studies found a significant positive correlation between them. For example, Quan et al. [18] found that adolescents who adopt positive coping style make full use of mobile phones to help them gain inspiration. Some studies showed no significant correlation between them. For example, Li et al. [20] found that adolescents who adopt positive coping style have more self-discipline, and mobile phones are only a tool for them to contact others and solve problems. Therefore, the first aim of the current meta-analysis was to investigate the relation between mobile phone addiction and coping style among adolescents.

As a secondary goal, we explored the potential moderators of effect sizes. Age, gender, publication type, year of publication and measurement tools were considered as potential moderators. First, some studies showed that there are differences in the degree of Internet addiction between middle school students and college students [21, 22]. Vocational college students (2-year or 3-year college students) have a stronger ability to resist pressure than undergraduates (4-year or 5-year college students) and are more inclined to choose positive coping styles, such as seeking help and problem solving, to cope with pressure [23–25]. Second, Dong et al. [26] found that when adolescents have problems such as mobile phone addiction, females are more flexible in changing their ways to adapt to pressure, while males, because they are not good at language expression and emotional communication, tend to choose negative coping styles such as escape in emotional regulation [27]. Third, generally, studies with significant results are often more likely to be published, so published journal articles may exaggerate the true relationship between variables [28]. Fourth, the emergence of mobile phone addiction has the characteristics of the times. With the popularization of the mobile internet and the emergence of diversified functions of smartphone, mobile phones have become an indispensable tool for modern people. Therefore, the relationship between adolescents’ mobile phone addiction and coping style may also change over time. Finally, as far as the measurement of mobile phone addiction is concerned, the contents of several kinds of measurement tools are not the same. For example, the Smartphone Addiction Scale for College Students (SAS-C) [29] is based on the existing research and combined with the research on the addiction to smartphone applications (apps); thus, adding the two factors of app use and app updates might better reflect the current level of adolescents’ mobile phone dependence. Similarly, in terms of the measurement of coping style, the Coping Style Questionnaire (CSQ) [30] and the Simplified Coping Style Questionnaire (SCSQ) [31] are widely used. The former divides coping styles into six categories: problem solving, self-blame, help seeking, fantasy, retreat and rationalization. The latter divides coping styles into two dimensions: positive and negative coping styles. Therefore, different tools for measuring coping style might also affect the relationship between adolescents’ mobile phone addiction and coping style.

In view of this, this study conducted a meta-analysis of the correlation between mobile phone addiction and coping style among adolescents, deeply investigated the correlation between them, and explored the moderators affecting the relationship so as to provide an objective basis and direction for the effective prevention and treatment of mobile phone addiction among adolescents.

## Methods

### Search strategy

A comprehensive search of Chinese and English literature was conducted to collect published studies related to mobile phone addiction and coping style at home and abroad. Two authors independently retrieved relevant studies from the China National Knowledge Infrastructure (CNKI), WANFANG DATA and Chongqing VIP Information Co., Ltd. (VIP), PubMed, Web of Science, Embase, and PsycINFO. Search terms used for mobile phones included “cell phone”, “mobile phone”, “smart phone”, “smartphone”, and “cellular phone”. Search terms used for addiction included “addiction”, “dependence”, “use”, “abuse”, “dependency”, “addicted to”, “overuse”, “problem use”, and “compensatory use”. Search terms used for coping style included “coping style”, “coping styles”, “coping mode”, “coping strategy”, and “coping strategies”. Other search terms included “problematic cell phone use”, “problematic mobile phone use”, “problematic smart phone use”, “problematic smartphone use”, and “problematic cellular use”. In addition, this study traced the references included in the identified studies and supplements as well as Chinese and English key words to identify relevant literature. The retrieval time spanned from the creation of the database to March 18, 2021.

### Inclusion and exclusion criteria

The inclusion criteria were as follows: (a) the study design was a cross-sectional survey; (b) a clear scale was used to assess mobile phone addiction and coping style; (c) the correlation coefficient between mobile phone addiction and positive coping style or negative coping style was reported, and if the correlation coefficient of the total score was not reported, the full factor correlation coefficient should be reported; (d) the subjects were healthy adolescents; and (e) both published articles and dissertations were included. The exclusion criteria were as follows: (a) studies not published in Chinese or English; (b) studies with the same data published repeatedly; (c) studies with apparent data mistakes; and (d) studies with samples containing individuals with physical diseases or mental disorders.

### Coding variables

The included articles were coded as follows: author, year of publication, region, sample size, gender, participant categories, publication type, correlation coefficient, mobile phone addiction scale and coping style scale (Table 1). For the input of the correlation coefficient, the coding standards were as follows: (a) If the correlation coefficient between coping style and mobile phone addiction is not reported but the values of *F*, *T* and *χ*^*2*^ are reported, they are transformed into the *r* value by the corresponding formula ($r=\sqrt{\frac{{t}^{2}}{{t}^{2}+df}}$, *df* = n_1_ + n_2_-2; $r= \sqrt{\frac{F}{F+{df}_{e}}}$; $r= \sqrt{\frac{\chi 2}{\chi 2 +N}}$) [32]. (b) The study effect size was encoded as an effect size according to the independent samples. If the study contained multiple independent samples, the article effect size was coded separately. The studies that examined the correlation between mobile phone addiction and positive coping style and those that examined the correlation between mobile phone addiction and negative coping style were coded. (c) If only the correlation coefficients of certain dimensions between mobile phone addiction and coping style were reported, the average of each dimension was taken before coding.

**Table 1:** Characteristics of the 33 studies included in the analysis

Name (year)	Region	Sample size	Female%	Subject type	Publication type	*R* (PCS and NCS)	Measurement tool	
							Mobile phone addiction	Coping style
Quan (2014) [18]	Western	546	0.55	2	Dissertation	0.12 and − 0.25	MPATS	CSQ
Xu (2014) [38]	Eastern	293	0.54	1	Journal	0.01 and 0.21	MPDI	CCSS-MSS
Wu (2015) [39]	Central	687	0.61	3	Dissertation	− 0.01 and 0.29	Self-compiled	SCSQ
Zeng (2015) [40]	Central	282	0.60	3	Dissertation	0.04 and 0.34	MPATS	CSQ
Chen (2015) [19]	Central	421	0.53	3	Journal	0.19 and − 0.17	MPAI	CSQ
Zhang (2015) [41]	Central	307	0.69	3	Journal	0.11 and 0.33	MPATS	SCSQ
Li (2016) [20]	N	696	0.71	3	Journal	− 0.03 and 0.31	MPAI	SCSQ
Zhang (2016) [42]	N	276	0.70	3	Journal	− 0.14 and 0.39	MPATS	SCSQ
Zu (2016) [16]	Central	548	0.60	3	Journal	− 0.01 and 0.32	MPATS	CSQ
Yan (2016) [43]	Eastern	404	0.41	2	Journal	N and 0.20	MPAI	CSQ
Wu (2016) [44]	Central	624	0.55	2	Dissertation	0.15 and 0.27	MPAI	HCSS-MSS
Chen (2017) [45]	Central	432	0.52	3	Dissertation	0.09 and 0.36	MPAI	SCSQ
Gao (2017) [46]	Western	488	0.21	2	Dissertation	0.11 and 0.36	MPAI	SCSQ
Wang (2017) [47]	Central	490	0.72	3	Dissertation	N and 0.35	SAS-C	SCSQ
Xia (2017) [48]	Central	330	0.64	3	Journal	− 0.17 and 0.34	MPATS	CSQ
Xin (2017) [49]	Central	630	0.44	1	Journal	− 0.13 and 0.27	MPDS-MSS	CCSS-MSS
Zeng (2018) [50]	Central	395	0.59	3	Dissertation	− 0.06 and 0.31	MPATS	SCSQ
He (2018) [17]	Central	490	0.72	3	Journal	N and 0.35	SAS-C	SCSQ
Liu (2018) [51]	Eastern	728	0.79	3	Journal	− 0.01 and 0.19	MPAI	WSCSQ
Xiong (2018) [52]	Central	359	0.60	3	Journal	N and 0.30	MPATS	SCSQ
Sun (2018) [53]	M	1041	0.44	1	Journal	− 0.13 and 0.35	SAS-C	CCSS-MSS
Xu (2018) [54]	Western	316	0.53	1	Journal	0.07 and N	MPAS	SCOPE
Zeng (2019) [55]	Eastern	2178	0.49	2	Journal	− 0.01 and 0.26	MPATS	SCSQ
Xu (2019) [56]	N	558	0.60	3	Journal	− 0.02 and 0.36	MPAI	SCSQ
Zhang (2019) [57]	Central	1774	0.86	3	Journal	− 0.01 and 0.20	MPAI	SCSQ
Hong (2019) [58]	Eastern	522	0.59	3	Journal	− 0.08 and 0.31	MPAI	CSQ
Han (2020) [59]	N	366	0.57	3	Dissertation	0.08 and 0.73	MPAI	SCSQ
He (2020) [60]	M	911	0.55	2	Journal	− 0.15 and 0.51	MPATS	SCSQ
Yuan (2020) [61]	Central	870	0.77	3	Dissertation	N and 0.38	TMD-C	SCSQ
Zheng (2020) [62]	N	418	0.64	3	Journal	− 0.24 and 0.23	SAS-CA	SCSQ
Liu (2020) [63]	Eastern	1169	0.44	3	Journal	0.03 and 0.43	MPAI	SCSQ
He (2020) [15]	M	604	0.56	1 and 3	Journal	N and 0.28	MPAI	SCSQ
Qiu (2021) [64]	Central	190	0.68	3	Journal	0.07 and 0.27	MPAI	SCSQ

*N* Not reported, *M* Mixed region, *1* Middle school students, *2* Vocational college students,*3*  Undergraduate, *PCS* Positive coping, *NCS* Negative coping style, *MPATS* Mobile Phone Addiction Tendency Scale, *MPDI* Mobile Phone Dependence Inventory, *MPAI* Mobile Phone Addiction Index, *SAS-C* Smartphone Addiction Scale for College Students, *MPDS-MSS* Mobile Phone Dependence Scale for Middle School Students, *MPAS* Smartphone addiction scale, *TMD-C* the Test of Mobile Phone Dependence for Chinese Adolescents, *SAS-CA* Smartphone Addiction Scale for Chinese Adults, *CSQ* Coping Style Questionnaire, *SCSQ* the Simplified Coping Style Questionnaire, *CCSS-MSS* Chen’s Coping Style Scale for Middle School Students, *HCSS-MSS* Huang’s Coping Style Scale for Middle School Students, *WSCSQ* Wang’s Simplified Coping Style Questionnaire, *SCOPE* the Student Coping Instrument

### Quality assessment

The literature quality evaluation was completed by two researchers independently. Any doubts or disagreement were resolved by centralized discussion (at least three people) or by soliciting the opinions of third-party experts. The nine-item Joanna Briggs Institution Critical Appraisal Checklist for Studies Reporting Prevalence Data was used to assess literature quality in this study [33]. The score for each item is zero (“no”, “unclear” or “not applicable”) or one (“Yes”), and the highest score is nine. Higher scores reflected better methodological quality.

### Data processing and analyses

Stata 16.0 software was used for meta-analysis, and the correlation coefficient *r* was used as the effect size in this study. Specifically, the *r* value was first converted to the corresponding Fisher’s *Z* value by using the Fisher transform, weighted based on the sample size with 95% confidence intervals: *Z* = 0.5*ln[(1 + *r*)/(1 − *r*)], where the variance of *Z* is V_*Z*_ = 1/n − 3 and the standard deviation of *Z* is SE_*Z*_ = square root of (1/n − 3). The evaluation criteria were as follows: when the correlation coefficient effect size *r* ≤ 0.10, it was a low correlation; when 0.10 < *r* < 0.40, the correlation was moderate; and when *r* ≥ 0.40, the correlation was high [34]. Publication bias was analysed by funnel plots and Egger’s linear regression test, and heterogeneity was examined with Cochran’s *Q* and *I*^*2*^ statistics. When the *Q* value was significant (*P* < 0.05) and *I*^*2*^ ≥ 75%, this indicated a high degree of heterogeneity in the study, and it was more reasonable to choose the random effects model; otherwise, the fixed effects model was chosen [35]. In addition, subgroup analysis and sensitivity analysis were necessary to investigate the sources of heterogeneity.

## Results

### Basic characteristics of the included studies and quality assessment

**Fig. 1:**
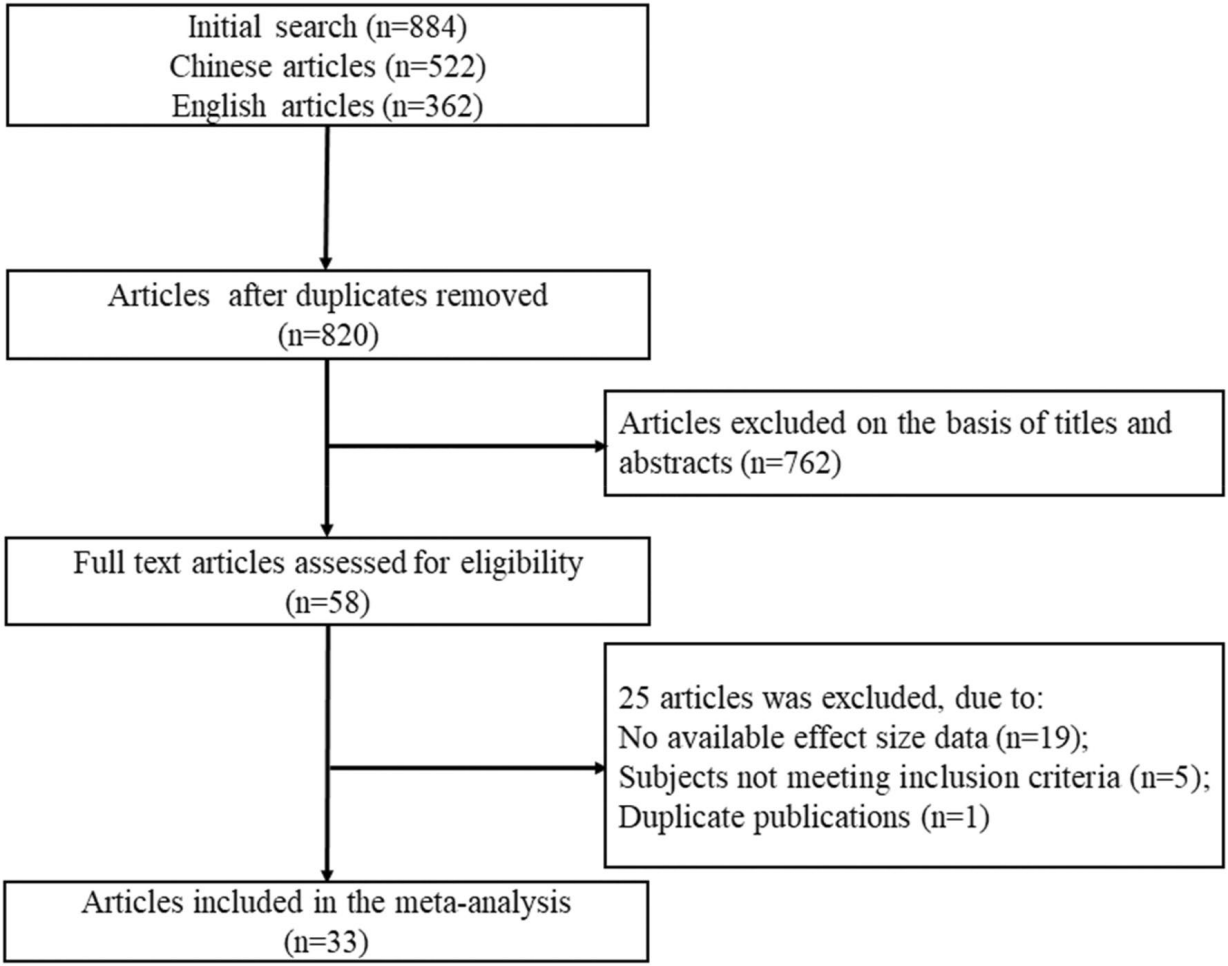
The flow chart of the study selection process

A total of 884 articles were initially retrieved in this study. After removing duplicates, the search produced 820 studies. A total of 762 studies were excluded based on titles and abstracts. Then, the full texts of 58 studies were assessed for eligibility, and 33 studies were ultimately included (see Fig. 1). Those included studies were published between 2014 and 2021. Among them, there were 10 dissertations and 23 journal articles. Collectively, 20,349 participants were enrolled in those studies, including 8318 males and 12,031 females. The largest sample size was 2178, and the smallest was 190. All studies used self-report scales to measure both mobile phone addiction and coping style. The most frequently used measures of mobile phone addiction were the Mobile Phone Addiction Index (MPAI) [36] and Mobile Phone Addiction Tendency Scale (MPATS) [37]. Similarly, the most common instruments used for assessing coping style were the Simplified Coping Style Questionnaire (SCSQ) [31] and the Coping Style Questionnaire (CSQ) [30] (Table 1). In general, the quality of the included studies was at a medium or high level. The results from the quality assessment of each study can be found in Additional file 1: Table S1.

### Effect size and heterogeneity test

A heterogeneity test was conducted on the included effect sizes, and the results showed that the *Q* values of positive and negative coping styles were 140.27 (*P* < 0.001) and 474.33 (*P* < 0.001), respectively, and the *I*^*2*^ values were 81.5% and 93.5%, respectively, both higher than the 75% rule proposed by Higgins et al. [65], indicating a high level of heterogeneity among studies. Therefore, the random effects model was selected for meta-analysis. The results also suggest that it is necessary to rationally explore the moderating variables that affect the relationship between them.

The random effects model showed no significant correlation between positive coping style and mobile phone addiction among adolescents and a moderate positive correlation between negative coping style and mobile phone addiction among adolescents (positive coping style: *r* =  − 0.02, 95% CI =  − 0.06 to 0.02, *P* > 0.05; negative coping style: *r* = 0.31, 95% CI = 0.26 to 0.36, *P* < 0.001) (Table 2).

**Table 2:** Effect size and its heterogeneity test and publication bias test

Outcome Variable	*k*	*N*	*r*	Heterogeneity test			Publication bias test			
				*Q*	*df*	*I*^*2*^	Egger’s intercept	*SE*	95%*CI*	*P*
Positive coping style	27	17,132	− 0.02	140.27**	26	81.5%	0.36	1.52	[− 2.77, 3.48]	0.82
Negative coping style	32	20,033	0.31**	474.33**	31	93.5%	1.13	2.49	[− 3.96, 6.22]	0.65

**P < 0.01

### Publication bias and sensitivity analysis

**Fig. 2:**
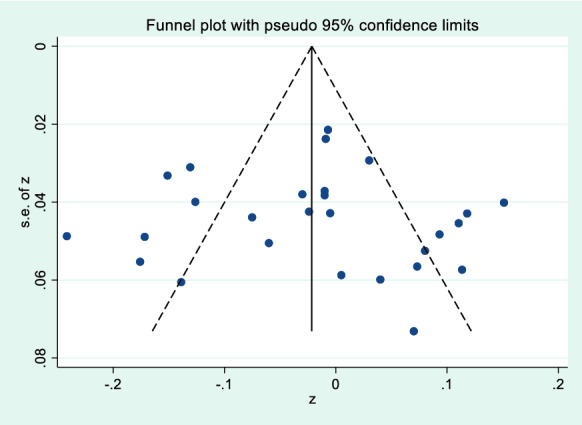
Funnel plot of the correlation of mobile phone addiction and positive coping style

**Fig. 3:**
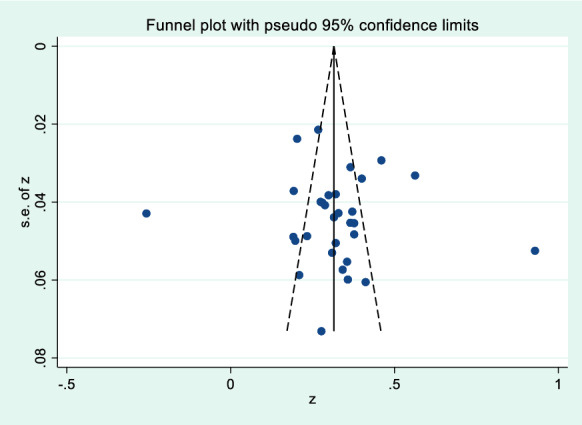
Funnel plot of the correlation of mobile phone addiction and negative coping style

First, the meta-analysis was tested by funnel plot for publication bias. Figures 2 and 3 show that the effect sizes of the relationship between mobile phone addiction and positive coping style and negative coping style of adolescents were basically evenly distributed on both sides of the overall effect sizes, indicating that the risk of publication bias was small in the study. Second, Egger’s linear regression tests found that the *P* values of positive coping style (*P* = 0.82) and negative coping style (*P* = 0.65) were greater than 0.05, which further indicated that there was no publication bias in this study, and the estimated results of meta-analysis were relatively reliable. The sensitivity analysis of the included literature was conducted by culling one by one, and the results showed little change, indicating that the results of this study were relatively stable.

### Moderating effect test

The heterogeneity of effects across studies was explored through moderator analysis. In this study, the moderating effect of 6 variables was tested: age, gender, publication type, year of publication, tool for measuring mobile phone addiction and tool for measuring coping style (Tables 3 and 4). The results showed that in terms of publication type, the effect of dissertations on mobile phone addiction and positive coping style among adolescents was significantly larger than that of journal articles (dissertations: *r* = 0.07, 95% CI = 0.02 to 0.12, *P* < 0.05; journal articles: *r* =  − 0.05, 95% CI =  − 0.09 to − 0.02, *P* < 0.001). In the tool for measuring mobile phone addiction, the SAS-C had the largest effect on mobile phone addiction and positive coping style among adolescents (*r* =  − 0.13, 95% CI =  − 0.19 to − 0.07, *P* < 0.001). For the year of publication, the time of publication significantly positively moderated the relationship between mobile phone addiction and negative coping style (*P* < 0.05). In the tool for measuring coping style, the SCSQ had the largest effect on adolescents’ mobile phone addiction and negative coping style (*r* = 0.37, 95% CI = 0.31 to 0.44, *P* < 0.001). However, the moderating effects of age and gender on adolescents’ mobile phone addiction and coping style were not significant (all *P* > 0.05). Therefore, the meta-analyses presented a high level of heterogeneity between studies, mainly stemming from the publication type, year of publication, tool for measuring mobile phone addiction and tool for measuring coping style.

**Table 3:** Coping style and mobile phone addiction: Univariate analysis of variance for moderator variables (categorical variables)

Moderators	Between-group effect (*Q*_*BET*_)	*k*	*N*	*r*	95%CI	Homogeneity test within each group (*Q*_*W*_)	*I*^*2*^
Positive coping style							
Participants type	0.50						
Middle school students		6	3635	0.01	[− 0.10, 0.12]	48.96***	89.8
Vocational college students		3	3392	− 0.02	[− 0.15, 0.11]	26.26***	92.4
Undergraduate		18	10,105	− 0.03	[− 0.07, 0.01]	64.73***	73.7
Proportion of women	0.67						
≥ 0.6		12	6800	− 0.04	[− 0.08, 0.01]	40.24***	72.7
< 0.6		15	10,736	− 0.01	[− 0.06, 0.05]	99.16***	85.9
Publication type	13.89***						
Journal		19	13,716	− 0.05	[− 0.09, − 0.02]	82.73***	78.2
Dissertation		8	3820	0.07	[0.02, 0.12]	17.66*	60.4
MPA measurement	14.60**						
MPATS		9	5773	− 0.03	[− 0.10, 0.04]	44.87***	82.2
MPAI		12	8372	0.02	[− 0.03, 0.06]	43.37***	74.6
SAS-C		1	1041	− 0.13	[− 0.19, − 0.07]	0.00	N/A
Others		5	2350	− 0.06	[− 0.17, 0.04]	24.88***	83.9
CS measurement	0.36						0.28
CSQ		6	2649	− 0.04	[− 0.14, 0.05]	29.90***	83.3
SCSQ		15	10,851	− 0.01	[− 0.06, 0.03]	68.53***	79.6
Others		6	3632	− 0.01	[− 0.10, 0.09]	40.68***	87.7
Negative coping style							
Participants type	1.82						
Middle school students		5	3076	0.31	[0.25, 0.36]	9.55*	58.1
Vocational college students		4	4039	0.19	[− 0.10, 0.49]	230.15***	98.7
Undergraduate		22	12,314	0.35	[0.29, 0.41]	215.06***	90.2
Proportion of women	0.01						
≥ 0.6		16	9009	0.32	[0.28, 0.36]	49.41***	69.6
< 0.6		16	11,024	0.32	[0.22, 0.42]	422.08***	96.4
Publication type	0.13						
Journal		22	14,853	0.31	[0.27, 0.36]	147.13***	85.7
Dissertation		10	5180	0.34	[0.18, 0.51]	326.61***	97.2
MPA measurement	3.69						
MPATS		10	6132	0.30	[0.16, 0.44]	240.01***	96.3
MPAI		14	8976	0.34	[0.25, 0.43]	212.90***	93.9
SAS-C		3	2021	0.37	[0.32, 0.41]	0.00	N/A
Others		5	2904	0.29	[0.22, 0.36]	13.28*	69.9
CS measurement	6.51*						
CSQ		7	3053	0.21	[0.04, 0.39]	144.03***	95.8
SCSQ		20	13,664	0.37	[0.31, 0.44]	246.85***	92.3
Others		5	3316	0.27	[0.20, 0.34]	14.62**	72.6

*P < 0.05, **P < 0.01, ***P < 0.001

**Table 4:** Univariate regression analysis of continuous variables (random effect model)

Moderators	*k*	*SE*	*t*	95%CI	*P*
Positive coping style (year)	27	0.01	− 1.08	[− 0.03, 0.01]	0.29
Negative coping style (year)	32	0.01	2.76	[0.01, 0.07]	0.01

## Discussion

The results showed that there was no significant correlation between mobile phone addiction and positive coping style (*r* =  − 0.02, *P* > 0.05), but there was a moderate positive correlation between mobile phone addiction and negative coping style (*r* = 0.31, *P* < 0.001). This suggests that adolescents who are more addicted to mobile phones tend to adopt negative coping styles such as avoidance, fantasy, and denial to maintain their inner balance when facing pressure, which is consistent with previous studies. He et al. [17] and Li et al. [66] found that adolescents with high levels of loneliness were not very sociable in daily life and were prone to feeling helpless, which made them more inclined to adopt negative coping styles such as avoidance and fantasy. Wu et al. [44] found that adolescents with negative coping tend to feel psychologically uncomfortable in the face of pressure and dilemma, and the convenience and entertainment of mobile phones can be used as a way to vent about their bad experience so as to temporarily ignore the distress caused by their problems in life. Moreover, according to the theories of compensatory internet use and coping and defence mechanisms, the more pressure adolescents feel, the more likely they are to use mobile phones to cope with the discomfort caused by that pressure [67, 68].

According to the results of the subgroup analyses, age did not have a moderating effect on the relationship between adolescents’ mobile phone addiction and coping style, perhaps because middle school students, vocational college students and undergraduates are all in a social environment where mobile networks and smartphones are widely available. Even for middle school students, approximately 80% own mobile phones, and nearly 40% of them use mobile phones without time limitations [69]. The external environment has the same influence on them. Therefore, there is little difference in coping styles when they encounter pressure or difficulties. Similarly, gender did not significantly moderate the relationship between adolescents’ mobile phone addiction and coping style, which was inconsistent with the results of some previous studies. Some studies showed that compared with females, males were more likely to adopt negative coping style, to experience more pressure, and to be addicted to mobile phones [15, 60]. Some studies also showed that females adopted more negative coping styles than males [64, 70, 71]. The reason for this difference might be that mobile phones meet different needs for males than for females.

Publication type significantly moderated the relationship between mobile phone addiction and coping style among adolescents. The correlation coefficients between adolescents’ mobile phone addiction and positive coping style reported in different types of literature were different, and the degree of correlation reported in dissertations was higher than that reported in journal articles. This result is inconsistent with the claims that in meta-analysis studies with publication bias, the effect size of journal articles is larger than that of dissertations [72]. This difference might be related to the quality of the study and the rigor of the review and to the expectation of a difference in the study results.

Year of publication significantly moderated the negative correlation between adolescents’ mobile phone addiction and negative coping style. The change trend of the correlation coefficient also indicated that the more recently the literature was published, the higher the correlation between mobile phone addiction and negative coping style among adolescents. This might be because with the popularity of mobile internet and smartphones, the multifunctional, interactive, entertainment and other characteristics of mobile phones not only cater to the psychological needs of adolescents, but also bring convenience and benefits to their daily life. Therefore, more and more adolescents spend most of their spare time on mobile phones [73], forming a habit of carrying or using mobile phones anytime and anywhere [74], which further increases the risk for mobile phone addiction, but also induce a series of anxiety, depression and other negative emotions [75]. When they face a difficult situation and lack sufficient support, they tend to adopt negative coping style to resist these negative emotions [67, 76]. Adolescents who adopt negative coping style for a long time are prone to mobile phone dependence[15].

The tools for measuring mobile phone addiction significantly moderated the relationship between adolescents’ mobile phone addiction and positive coping style but not the relationship between this addiction and negative coping style. The tools for measuring coping style significantly moderated the relationship between adolescents’ mobile phone addiction and negative coping style, but the moderating effect on the relationship between this type of addiction and positive coping style was not significant. First, as far as the tools for measuring mobile phone addiction are concerned, the correlation between mobile phone addiction and positive coping style among adolescents reported by the SAS-C was the highest. A possible reason is that compared with other scales, the SAS-C was compiled for college students in 2014, based on the existing studies and compiled in conjunction with research on smartphone app addiction [29], so it better reflects the current level of adolescents’ mobile phone dependence. Second, as far as the tools for measuring coping style are concerned, the effect of mobile phone addiction and negative coping style among adolescents reported by the SCSQ was the highest, which might be caused by the different dimensions subdivided by each scale. The SCSQ divided various coping styles into positive and negative dimensions according to their common characteristics, which were stable and balanced to a certain extent. However, the CSQ and the scales compiled by other scholars have problems such as the factor attribution of some items being unstable and unbalanced. Therefore, the SCSQ can better reflect the relationship between adolescents’ mobile phone addiction and negative coping style than other scales can.

## Limitations and prospects

Previous studies on the relationship between adolescents’ mobile phone addiction and coping style have been inconsistent. In this study, the meta-analysis method was used to investigate the relationship between adolescents’ mobile phone addiction and coping style, clarifying the controversy about the size and direction of the correlation between them in empirical studies. However, there are some limitations of this study. First, the studies included in the meta-analysis mainly focused on adolescents; research can be further expanded in the future to explore whether there are differences in the relationship between mobile phone addiction and coping style among different age groups. Second, this study only focused on the influence of some moderator variables on the relationship between mobile phone addiction and coping style, and other potential moderator variables should be analysed in the future, such as personality and cultural background. Finally, the studies included in this meta-analysis were cross-sectional, and longitudinal studies can be used in the future to establish the direction(s) of causality for the relationship between coping style and mobile phone addiction.

## Conclusion

There was no significant correlation between adolescents’ mobile phone addiction and positive coping style, while there was a moderate positive correlation between this type of addiction and negative coping style. Thus, adolescents with negative coping style had a higher degree of dependence on mobile phones. Publication type and mobile phone addiction measurement tools significantly moderated the relationship between adolescents’ mobile phone addiction and positive coping style. Year of publication and coping style measurement tools significantly moderated the relationship between adolescents’ mobile phone addiction and negative coping style, but the correlation between their mobile phone addiction and coping style was not moderated by age or gender. In the future, longitudinal studies should be carried out to better investigate the dynamic changes in the relationship between adolescents’ mobile phone addiction and coping style.

## Supplementary Information

**Additional file 1: Table S1.** Quality assessment for the 33 studies in the current meta-analysis.

## Data Availability

The datasets used and/or analysed during the current study are available from the corresponding author on reasonable request.

## Abbreviations

MPATS: Mobile Phone Addiction Tendency Scale
MPDI: Mobile Phone Dependence Inventory
MPAI: Mobile Phone Addiction Index
SAS-C: Smartphone Addiction Scale for College Students
MPDS-MSS: Mobile Phone Dependence Scale for Middle School Students
MPAS: Smartphone addiction scale
TMD-C: The Test of Mobile Phone Dependence for Chinese Adolescents
SAS-CA: Smartphone Addiction Scale for Chinese Adults
CSQ: Coping Style Questionnaire
SCSQ: The Simplified Coping Style Questionnaire
CCSS-MSS: Chen’s Coping Style Scale for Middle School Students
HCSS-MSS: Huang’s Coping Style Scale for Middle School Students
WSCSQ: Wang’s Simplified Coping Style Questionnaire
SCOPE: The Student Coping Instrument
