# Supplementary material for: The correlation between mobile phone addiction and coping style among Chinese adolescents: a meta-analysis
Source: Child Adolesc Psychiatry Ment Health. 2021 Oct 15;15:60. doi: 10.1186/s13034-021-00413-2 (PMC8520246; doi:10.1186/s13034-021-00413-2)
Supplement: Supplementary file 1 — Additional file 1: Table S1. Quality assessment for the 33 studies in the current meta-analysis. [file 13034_2021_413_MOESM1_ESM.docx]

Table S1.Quality assessment for the 33 studies in the current meta-analysis.

| Study | Quality Item | | | | | | | | | |
| --- | --- | --- | --- | --- | --- | --- | --- | --- | --- | --- |
|  | Item1 | Item2 | Item3 | Item4 | Item5 | Item6 | Item7 | Item8 | Item9 | Total |
| Quan (2014) [18] | Y | U | Y | Y | Y | Y | Y | Y | N | 7 |
| Xu (2014) [38] | Y | Y | Y | N | Y | Y | Y | Y | Y | 8 |
| Wu (2015) [39] | Y | Y | Y | Y | Y | Y | Y | Y | Y | 9 |
| Zeng (2015) [40] | Y | U | Y | Y | Y | Y | Y | Y | Y | 8 |
| Chen (2015) [19] | Y | Y | Y | Y | Y | Y | Y | Y | Y | 9 |
| Zhang (2015) [41] | Y | Y | Y | Y | Y | Y | U | Y | Y | 8 |
| Li (2016) [20] | Y | Y | Y | N | Y | Y | U | Y | Y | 7 |
| Zhang (2016) [42] | Y | Y | Y | N | Y | Y | Y | Y | Y | 8 |
| Zu (2016) [16] | Y | U | Y | Y | Y | Y | Y | Y | Y | 8 |
| Yan (2016) [43] | Y | Y | Y | Y | Y | Y | Y | Y | Y | 9 |
| Wu (2016) [44] | Y | Y | Y | Y | Y | Y | N | Y | Y | 8 |
| Chen (2017) [45] | Y | Y | Y | Y | Y | Y | N | Y | Y | 8 |
| Gao (2017) [46] | Y | Y | Y | Y | Y | Y | Y | Y | Y | 9 |
| Wang (2017) [47] | Y | Y | Y | Y | Y | U | Y | Y | Y | 8 |
| Xia (2017) [48] | Y | Y | Y | Y | Y | Y | Y | Y | Y | 9 |
| Xin (2017) [49] | Y | Y | Y | Y | Y | Y | Y | Y | Y | 9 |
| Zeng (2018) [50] | Y | Y | Y | Y | Y | Y | Y | Y | Y | 9 |
| He (2018) [17] | Y | Y | Y | Y | Y | N | N | Y | Y | 7 |
| Liu (2018) [51] | Y | Y | Y | Y | Y | Y | N | Y | Y | 8 |
| Xiong (2018) [52] | Y | Y | Y | Y | Y | N | N | Y | Y | 7 |
| Sun (2018) [53] | Y | U | Y | Y | Y | Y | Y | Y | U | 7 |
| Xu (2018) [54] | Y | U | Y | Y | Y | Y | Y | Y | Y | 8 |
| Zeng (2019) [55] | Y | Y | Y | Y | Y | N | N | Y | Y | 7 |
| Xu (2019) [56] | Y | N | Y | N | Y | N | Y | Y | Y | 6 |
| Zhang (2019) [57] | Y | Y | Y | Y | Y | Y | Y | Y | U | 8 |
| Hong (2019) [58] | Y | Y | Y | Y | Y | Y | Y | Y | Y | 9 |
| Han (2020) [59] | Y | Y | Y | N | Y | Y | Y | Y | N | 7 |
| He (2020) [60] | Y | N | Y | Y | Y | Y | Y | Y | Y | 8 |
| Yuan (2020) [61] | Y | Y | Y | Y | Y | Y | N | Y | Y | 8 |
| Zheng (2020) [62] | Y | U | Y | N | Y | N | Y | Y | Y | 6 |
| Liu (2020) [63] | Y | Y | Y | Y | Y | Y | Y | Y | Y | 9 |
| He (2020) [15] | Y | Y | Y | Y | Y | N | Y | Y | U | 7 |
| Qiu (2021) [64] | Y | Y | Y | Y | Y | Y | N | Y | Y | 8 |

*Abbreviations:* Y, yes; N, No; U, unclear.
